# The epidemiology of pre-hospital potential spinal cord injuries in Victoria, Australia: a six year retrospective cohort study

**DOI:** 10.1186/s40621-016-0089-0

**Published:** 2016-10-17

**Authors:** Ala’a O. Oteir, Karen Smith, Johannes U. Stoelwinder, Shelley Cox, James W. Middleton, Paul A. Jennings

**Affiliations:** 1 Department of Community Emergency Health and Paramedic Practice, Monash University, Building 3, 270 Ferntree Gully Road, Notting Hill, VIC 3168 Australia; 2 Research and Evaluation, Ambulance Victoria, Melbourne, Victoria Australia; 3 Department of Epidemiology and Preventive Medicine, Monash University, Melbourne, Victoria Australia; 4 Department of Emergency Medicine, University of Western Australia, Perth, Western Australia Australia; 5 John Walsh Centre for Rehabilitation Research, Kolling Institute, Northern Sydney Local Health District, St Leonards and Sydney Medical School-Northern, The University of Sydney, New South Wales, Australia; 6 Ambulance Victoria, Melbourne, Victoria Australia; 7 Emergency and Trauma Centre, The Alfred Hospital, Melbourne, Victoria Australia; 8 College of Health and Biomedicine, Victoria University, Melbourne, Victoria Australia

**Keywords:** Pre-hospital, Emergency Medical Services, Potential SCI, Suspected SCI, Traumatic SCI, TSCI, Epidemiology, Trauma

## Abstract

**Background:**

Traumatic Spinal Cord Injury (TSCI) is relatively uncommon, yet a devastating and costly condition. Despite the human and social impacts, studies describing patients with potential TSCI in the pre-hospital setting are scarce. This paper aims to describe the epidemiology of patients potentially at risk of or suspected to have a TSCI by paramedics, with a view to providing a better understanding of factors associated with potential TSCI.

**Methods:**

This is a retrospective cohort study of all adult patients managed and transported by Ambulance Victoria (AV) between 01 January 2007 and 31 December 2012 who, based on meeting pre-hospital triage protocols and criteria for spinal clearance, paramedic suspicion or spinal immobilisation, were classified to be at risk of or suspected to have a TSCI. Data was extracted from the AV data warehouse, including demographic details, trauma aetiology, paramedic assessment, management and other event characteristics.

**Results:**

A total of 106,059cases were included in the study, representing 2.3 % of all emergency transports by AV. Subjects had a median age of 51 years (interquartile range; 29–78) and 52.4 % were males (95 % CI 52–52.7). Males were significantly younger than females (M: 43 years [26–65] vs. F: 64 years [36–84], *p* =0.001). Falls and traffic accidents were the leading causes of injuries, comprising 46.9 and 39.4 % of cases, respectively. Other causes included accidents due to sport, animals, industrial work and diving, as well as violence and hanging. 29.9 % of patients were transported to a Major Trauma Service (MTS). A proportion of 48.8 % of the study population met the Pre-hospital Major Trauma criteria.

**Conclusion:**

This is the first study to describe the epidemiology of potential TSCI in Australia and is based on a large, state-wide sample. It provides background knowledge and a baseline for future research, as well as a reference point for future in policy. Falling and traffic related injuries were the leading causes of potential SCI. Future research is required to identify the proportion of confirmed TSCI among the potentials and factors associated with TSCI in prehospital settings.

## Background

Traumatic spinal cord injury (TSCI) is a relatively rare, but devastating injury that often leads to long-term disabilities. It not only affects the patient’s functioning, participation in work, health and quality of life, but also places a financial burden on the patient’s family, the community and the health care system (Pickett et al. 2006; Kattail et al. 2009). The global incidence of TSCI has been reported to vary between 9.2 and 246 cases per million persons per year with prevalence ranging from 236 to 1,298 per million population (Furlan et al. 2013). In Australia, there are approximately 300 new cases of TSCI admitted to specialised spinal cord injury units each year (Middleton et al. 2012; Norton 2007). The lifetime cost for a person with paraplegia is estimated at $5.0 million and at $9.5 million for tetraplegia (Economics A 2009). Previous studies have shown that TSCI predominantly occurs in males and is due to a variety of causes such as traffic accidents, falls, sporting accidents and violence (Pickett et al. 2006; Kattail et al. 2009; Middleton et al. 2012; Norton 2007; Sekhon and Fehlings 2001; Oteir et al. 2014).

Pre-hospital care providers are often the first healthcare professional to assess and manage patients who have traumatic injuries. As they do not have the tools to definitively diagnose TSCI patients, including x-ray, CT or MRI scan machines, they rely on available pre-hospital spinal immobilisation guidelines, as well as their judgment to identify patients who are at risk or most likely to have a TSCI; known as potential TSCI patients.

Despite the importance of correctly identifying patients with TSCI or unstable spinal fractures and the possibility for developing TSCI with injudicious management, studies describing patients with potential TSCI identified in the pre-hospital setting are scarce. The majority of studies have described the development and utility of spinal clearance criteria, tools or methods of spinal immobilisation (Vaillancourt et al. 2011; Hauswald et al. 1998; Kwan et al. 2001). This paper aims to describe the epidemiology of patients who were potentially at risk of or suspected to have a TSCI, with a view to providing a better understanding of potential factors leading to confirmed TSCI.

## Methods

### Study design

This is a retrospective cohort study of all adult patients with a potential SCI who were managed and transported by AV between 01 January 2007 and 31 December 2012.

### Setting

Ambulance Victoria is the sole provider for Emergency Medical Services (EMS) in the state, providing an emergency medical response to more than 5.7 million people across an area of more than 227,000 km^2^ (Ambulance Victoria 2014). Ambulance Victoria operates as a two-tiered emergency medical response system, which includes advanced life support (ALS) paramedics and mobile intensive care ambulance (MICA) paramedics. MICA paramedics can perform intubation and administer a wider range of medications than ALS paramedics (Cox et al. 2011; Ambulance Victoria 2014). In the 2013-14 financial year, AV responded to 844,061 ambulance requests, including emergency (552,268; 65.4 %) and non-emergency (291,793; 34.6 %) cases via both road and air ambulances (Ambulance Victoria 2014-2015).

### Data sources

Pre-hospital data was recorded via VACIS, an in-field electronic data capture system linked to an integrated data warehouse (Ambulance Victoria 2014-2015).

Relevant data items were extracted from the AV data warehouse, including demographic details, trauma aetiology, paramedic assessment, management and other event characteristics.

### Eligibility criteria, sources and methods of selection of participants

Patients were eligible to be in the study if they met the AV Pre-hospital Potential Major Trauma (PMT) criteria (Ambulance Victoria 2014), AV spinal immobilisation criteria (Ambulance Victoria 2014), were reported by paramedics as having a suspected TSCI or received spinal immobilisation and were transported to the hospital.

This study included two major groups of patients; patients at risk of having a TSCI based on pre-specified criteria and/or patients with suspected TSCI as reported by paramedics. This categorisation, is not mutually exclusive, was used to maximise the capture all potential SCI.

Patients at risk of TSCI, according to AV Clinical Practice Guideline (CPGs), are trauma patients who meet the Pre-hospital Potential Major Trauma Criteria (Ambulance Victoria 2014), spinal immobilisation criteria (Ambulance Victoria 2014), or patients who had a mechanism of injury that has a potential to cause a TSCI (Ambulance Victoria 2014).

Patients who met the PMT criteria, in this study, were classified under three categories: 1. actual time critical, which includes trauma patients with aberrant vital signs; 2. emergent time critical, which is based on the pattern of injury; or, 3. potential time critical, which includes patients who sustained a mechanism of injury (MOI) known to be associated with serious injury and one or more of the following: co-morbid disease, is aged greater than 55 years or is pregnant. Examples of positive MOIs include such mechanisms as: falls from a height >3 m, pedestrian impact, and ejection from a vehicle (Ambulance Victoria 2014). These categories were derived from relevant VACIS variables recorded by paramedics, and for the purpose of this study were coded in a hierarchical order (actual, emergent then potential), even though patients may meet more than one of the criteria.

Furthermore, patients who meet any of AV’s spinal immobilisation criteria (which are based on modified Canadian C-spine rules (Stiell et al. 2001; Stroh and Braude 2001) and Nexus criteria (Hoffman et al. 1992, 2000; Domeier et al. 2005) are considered at risk of TSCI and should recieve spinal precautions and be immobilised. For the purpose of this study, trauma patients met the spinal immobilisation criteria if they met any of the following criteria (Ambulance Victoria 2015):

- Older than 55 years old
- A co-morbidty including muscular weakness and/or a bone disease
- Unconsciousness, altered conscious state (GCS <15) or period of loss of consciousness
- Drug or alcohol affected
- Significant distracting injury
- Neurological deficit
- Spinal pain and/or tenderness

Furthermore, the second group of patients that were included in the study were suspected TSCI patients. This group included patients who were explicitly reported as “suspected SCI” or SCI in the electronic patient care reports (ePCR) or implicitly suspected to have a TSCI if the paramedics applied spinal immobilisation or transported the patients directly to the SCI unit due to the patient’s condition. Not all of these patients met the PMT criteria, which is designed to detect major trauma.

An electronic data filter was used to identify patients meeting inclusion/exclusion criteria. Patients were excluded if they were deceased at the scene, were less than 16 years old, had an interfacility transport only, or had a non-traumatic aetiology. Case descriptions (free text section of the patient care record) were also reviewed to exclude cases due to non-traumatic aetiologies or cases that did not meet inclusion criteria. Cases with insufficient pre-hospital data was also excluded.

### Statistical analysis

Continuous data was summarised as medians and interquartile ranges (IQRs) and categorical data was summarised as counts and percentages. Mann-Whitney U tests for continuous data was used to compare medians between groups. Chi square tests for categorical variables were used to compare proportions across groups. All reported *p*-values are two-sided and a value less than 0.05 were considered statistically significant. All statistical analyses were performed using STATA (version 12.1 Stata Corporation, College Station, TX, USA). The incidence rate was calculated by dividing the number of cases for each age group by the count of population per age group for the four most common injury causes, and reported per million population.

## Results

### Participants and cause of injury

**Fig. 1:**
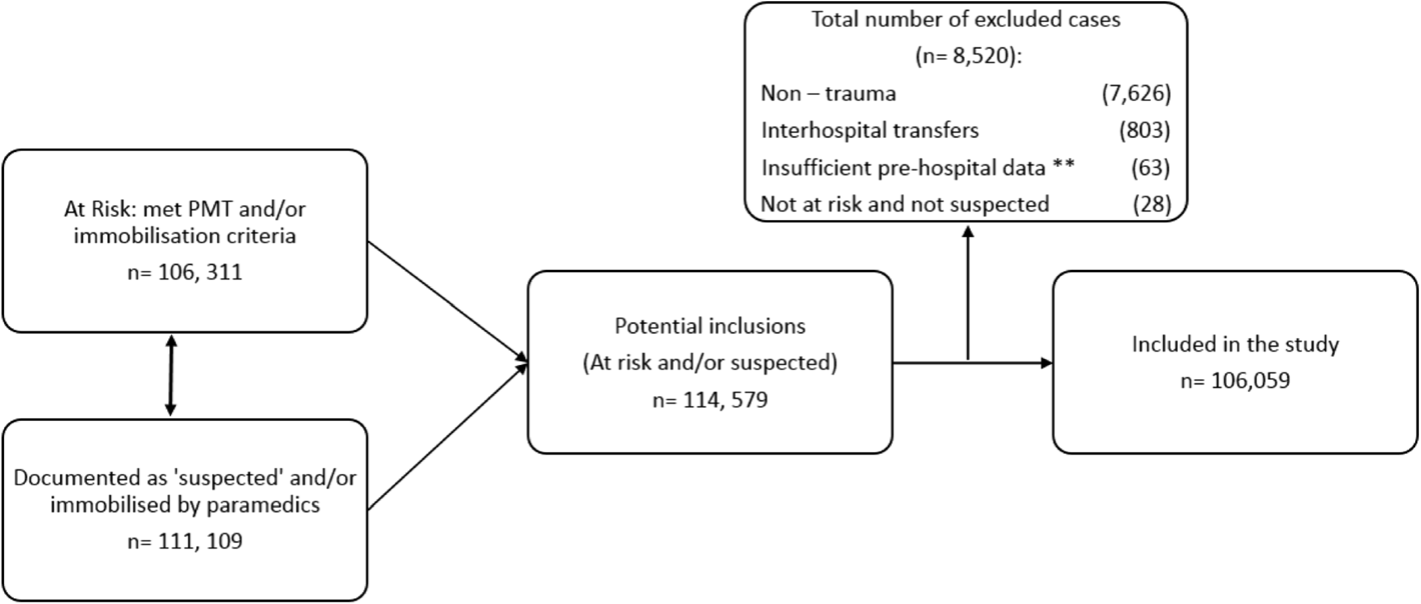
Inclusion flowchart of patients with potential traumatic spinal cord injury in Victoria, Australia (2007–2012). Note: initial categorisation is not mutually exclusive. ** insufficient pre-hospital data includes patient demographics, cause of injury, date and time components, immobilisation, destination and vital signs

During the study period, 114,579 patients were considered for eligibility. Of these, a total of 106,059(92.6 %) cases with traumatic injuries were included in the study (Fig. 1).

Table 1 summarises the patient demographics. The median age of all patients was 51 years (IQR; 29–78) and 52.4 % (95 % CI: 52.1–52.7) of patients were male. Male patients were significantly younger than female patients (M: 43 years [26–65] vs F: 64 years [36–84], *p* =0.001). The four most common causes of potential TSCI were falls, traffic crashes, violence and sporting related incidents.

**Table 1:** Patient demographics and event characteristics for potential TSCI in Victoria, Australia (2007–2012)

Characteristic	*N* = 106059
Age	
Median (years (IQR))	51 (29–78)
Min, Max (years)	16, 107
	N (%; 95%CI)
Sex	
Male	55561 (52.1; 52.1–52.7)
Female	50498 (47.6; 47.3–47.9)
Cause of injury	
Falls	49710 (46.9; 46.6–47.2)
Falls (NFS)	32608 (30.7; 30.5–31.0)
Falls <3 m	14739 (13.9; 13.7–14.1)
Falls >3 m	2363 (2.2; 2.1–2.3)
Traffic	41807 (39.4; 39.1–39.7)
MVCs	25573 (24.1; 23.9–24.3)
Motorcycle	5458 (5.1; 5.0–5.3)
Bicycle	4132 (3.9; 3.8–4.0)
Vehicle related	3605 (3.4; 3.3–3.5)
Pedestrian	3039 (2.9; 2.8–3.0)
Violence	7098 (6.7; 6.5–6.8)
Blunt	6721 (6.4; 6.3–6.6)
Penetrating	374 (0.4;–0.3–0.9)
Both	3 (0.05;–0.01–0.1)
Sporting	2920 (2.8; 2.7–2.9)
Struck by object	1769 (1.7; 1.6–1.7)
Animal related (mainly fall from horses)	1573 (1.5; 1.4–1.6)
Others	1182 (1.1; 1.1–1.2)

Note: *N* number of cases, *IQR* interquartile range, *3 m* three meters, *MVCs* motor vehicle crashes, *NFS* not further specified (but mainly due to slip or trip), *CI* confidence interval, *others* hanging, diving and industrial injuries

Table 2 summarises the number of males and females in each age group. The largest age group was patients aged between 16 and 24 years comprising 18 % (95 % CI: 17.8–18.3) of our population; followed by the eldest age group (>85 years old), who contributed 14.9 % (95 % CI; 14.7–15.1). Females account for the majority (63.9 %; 95 % CI: 63.4–64.4) of patients 65 years and older, whereas males comprise larger proportions than females in the remaining age groups (<65 years).

**Table 2:** Patients with potential TSCI, by sex and age group, in Victoria, Australia (2007–2012)

Age group (years)	Male *n* = 55559 (%; 95 % CI)	Female *n* = 50497 (%; 95 % CI)	Total *n* = 106056(%; 95 % CI)
16–24	12226 (22.0; 21.7–22.4)	6905 (13.7; 13.4–14.0)	19131 (18.0; 17.8–18.3)
25–34	9157 (16.5; 16.2–16.8)	5098 (10.1; 9.8–10.4)	14255 (13.4; 13.2–13.6)
35–44	7878 (14.2; 13.9–14.5)	4618 (9.1; 8.9–9.4)	12496 (11.8; 11.6–12.0)
45–54	6727 (12.1; 11.8–12.4)	4578 (9.1; 88.9–94.0)	11306 (10.7; 10.5–10.8)
55–64	5451 (9.8; 9.6–10.1)	4284 (8.4; 8.2–8.7)	9735 (9.2; 9.0–9.4)
65–74	4305 (7.8; 7.5–8.0)	4510 (8.9; 8.7–9.2)	8815 (8.3; 8.1–8.5)
75–84	5420 (9.8; 9.5–10.0)	9088 (18.0; 17.7–18.3)	14508 (13.7; 13.5–13.9)
>85	4395 (7.9; 7.7–8.1)	11416 (22.6; 22.2–23.0)	15811 (14.9; 14.7–15.1)

Chi square test = 7,900; *p* < 0.001; *CI* confidence interval, *TSCI* traumatic spinal cord injury

Table 3 outlines the major causes of potential SCI by age group. It shows that the majority of falls occur among elderly age groups (>65 years). Whereas, traffic related accidents mainly occur in the youngest age group (<24 years).

**Table 3:** Major causes of potential TSCI by age group in Victoria, Australia (2007–2012)

Age group (years)	Falls *n* = 49710 (%; 95 % CI)	Traffic *n* = 41805 (%; 95 % CI)	Other *n* = 14541 (%; 95 % CI)	Total *n* = 106056 (%; 95 % CI)
16–24	2604 (5.2; 5.0–5.4)	11090 (26.5; 26.1–26.9)	5437 (37.4; 36.6–38.1)	19131 (18.0; 17.8–18.3)
25–34	2368 (4.8; 4.6–5.0)	8580 (20.5; 20.1–20.9)	3307 (22.8; 22.1–23.4)	14255 (13.4; 13.2–13.6)
35–44	2890 (5.8; 5.6–6.0)	7060 (16.9; 16.5–17.2)	2546 (17.9; 16.9–18.1)	12496 (11.8; 11.6–12.0)
45–54	3781 (7.6; 7.4–7.8)	5847 (14.0; 13.7–14.3)	1677 (11.5; 11.0–12.1)	11306 (10.7; 10.5–10.8)
55–64	4646 (9.3; 9.1–9.6)	4207 (10.1; 9.8–10.4)	882 (6.1; 5.7–6.5)	9735 (9.2; 9.0–9.4)
65–74	5926 (11.9; 11.6–12.2)	2541 (6.1; 5.8–6.3)	348 (2.4; 2.1–2.6)	8815 (8.3; 8.1–8.5)
75–84	12475 (25.1; 24.7–25.5)	1819 (4.4; 4.2–4.5)	214 (1.5; 1.3–1.7)	14508 (13.7; 13.5–13.9)
>85	15020 (30.2; 29.8–30.6)	661 (1.6; 1.5–1.7)	130 (0.9;0.7–1.0)	15811 (14.9; 14.7–15.1)

*TSCI* traumatic spinal cord injury, *CI* confidence interval; Chi square test = 44,000; *p* < 0.001

### Cause of injury and incidence rates and patterns

**Fig. 2:**
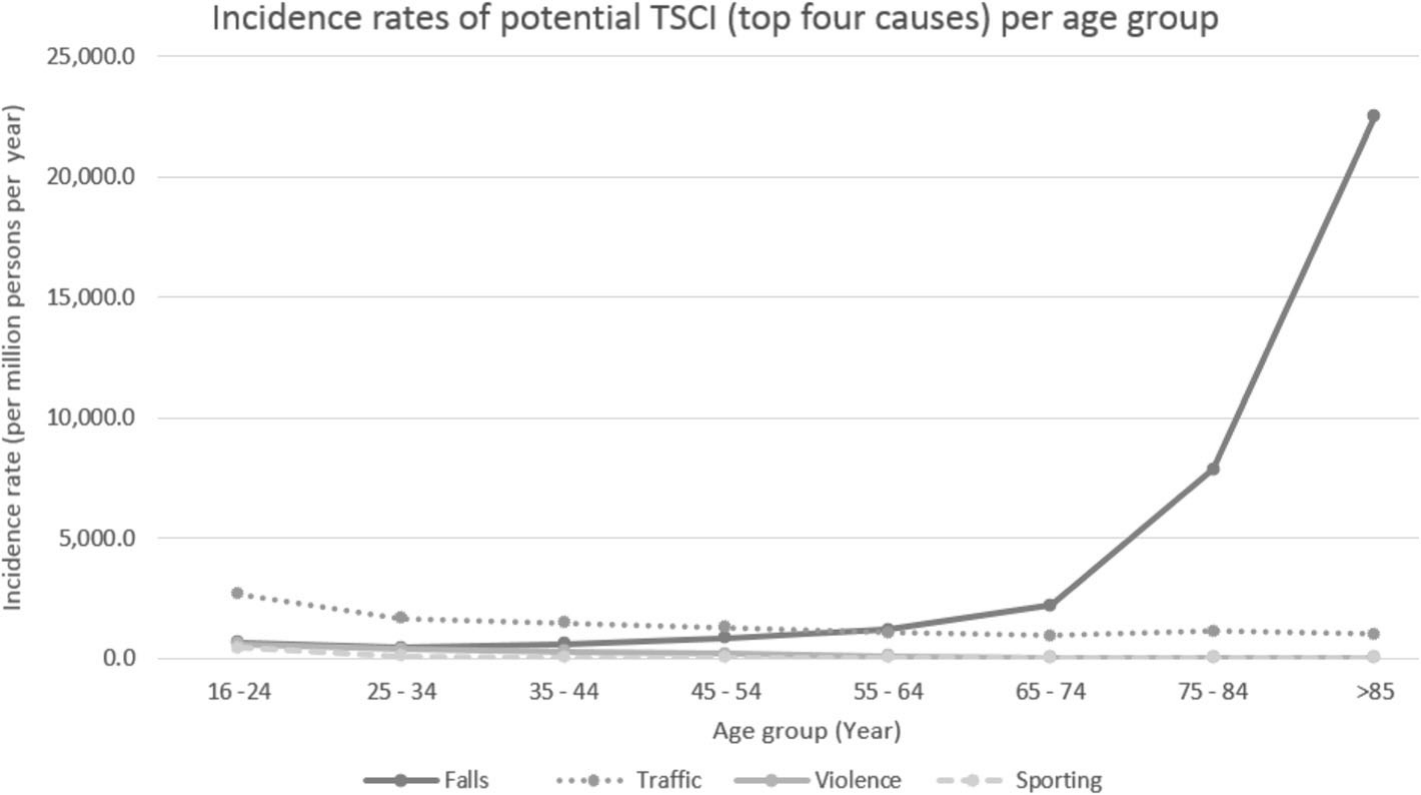
Incidence rates of potential traumatic spinal cord injuroes (top four causes) by age group in Victoria, Australia (2007–2012)

The average incidence for different types of injury varied over the study period, ranging from 4 (diving incidents) to 1,894 (falls) per million persons per year. A continuous increase was observed in falls rates, ranging from 1,033 in 2007 to 2,623 per million persons per year in 2012. Figure 2 outlines the incidence rates of the top four injuries per million persons per year by age categories.

#### Falls

Falls (46.9 %; 95 % CI: 46.6–47.2) were the leading cause of potential TSCI, with an average (standard deviation(SD)) incidence rate of 1,894 (580.1) per million persons per year. Falls increased with age, ranging from 620 in patients 16–24 years to 22,556 per million persons per year in patients older than 85 years (Fig. 2). The majority of falls were among females (58.2 %; 95 % CI: 57.7–58.6), with a higher median [IQR] age compared to males (F: 81 [66–88] vs M: 68 [47–82], *p* < 0.001). More than half of the falls occurred among patients who were older than 75 years (55.3 %) and occured from a standing level due to a slip or trip (65.6 %). Falls from less than three meters were most frequently observed among females older than 75 years (31.5 %; 4,647/14,739), whereas, high falls (>3 m), i.e., from ladders and roofs, mainly occurred among males aged between 45 and 64 years (27.9 %).

### Traffic related

Traffic related injuries were the second most frequent cause (39.4 %; 95 % CI: 39.1–39.7) of potential TSCI with a mean (SD) incidence rate of 1,602 (218) per million persons per year. Males comprised 57.7 % (95 % CI: 57.2–58.2) with a slightly lower median (IQR) age than females (M: 35 [24–50] vs. F: 38 [24–55], *p* <0.001). As shown in Fig. 2, there is a slight decreasing trend in incidence as age increases.

Sixty-one percent (95 % CI: 60.7–61.6) of traffic related events were due to motor vehicle collisions (MVCs), with the largest proportion being among the youngest age group (27.9 %, 7,142/25,572). Whilst the number of incidences overall were similar between males and females, the crude incidents of MVCs were more prevalent in males younger than 34 years and among females older than 45 years.

The large majority of motorcycle and bicycle collisions involved males; 83.4 % (95 % CI: 82.4–84.4) and 75.8 % (95 % CI: 74.5–77.1), respectively. While the younger people (16–24 years) involved in motorcycle collisions comprised 28.6 % (95 % CI: 27.4–29.8) of these events, bicycle collisions occurred most frequently in the age group 25–34 years (23.5 %; 95 % CI: 22.2–24.8). Males involved in motorcycle collisions were generally younger than females, but were older in bicycle collisions (F: 34 years [27–48] vs. M: 40 years [29–52], p <0.001). In addition, pedestrian injuries were also most common among young adults (16–24 year, 25.5 %; 95 % CI: 24.0–27.1) primarily involving males (56.4 %; 95 % CI: 52.9–59.9).

### Remaining causes of injury

Violence, sport related, animal, crush, industrial accidents, diving, hanging and other causes accounted for only 13.7 % (95 % CI 13.9–13.9) of potential TSCI in this cohort. The majority of incidents were amongst the youngest group (16–24 years), except for hanging (25–44 years). Whilst most of these injuries were more prevalent among males, there were more females involved in animal related injuries.

### Pre-hospital Potential Major Trauma (PMT) Criteria and transport destination

Pre-hospital care providers use PMT criteria to triage patients with severe injuries. Patients who meet these criteria should be transported to a Major Trauma Service (MTS) if transport achievable within 45 min and considered at risk of TSCI, and for this reason the description of PMT components and transport destinations are provided in this section. Of 106,060 potential TSCI patients, 48.8 % (95 % CI: 48.5–49.1) met AV’s PMT criteria. This included 20.8 % (95 % CI: 20.5–21.2) of cases with specific anatomical injuries that are outlined in the PMT criteria and known to be associated with the risk of the patients’ condition deteriorating. Also, 72.1 % (95 % CI: 71.7–72.5) of cases had aberrant vital signs, and 7.0 % (95 % CI: 6.8–7.3) were due to a mechanism of injury combined with a comorbidity, pregnancy or age >55 years (Table 4).

**Table 4:** Patients who met the Pre-hospital Major Trauma Criteria in Victoria, Australia (2007–2012)

Pre-hospital Potential Major Trauma (PMT)	51788 (100 %; 95 % CI)
Actual time critical based on aberrant vital signs	10797 (20.9; 20.5–21.2)
Emergent time critical based on anatomic Injuries	37343 (72.1; 71.7–72.5)
Potential time critical based on mechanism of injury + modifier^a^	3648 (7.0; 6.8–7.3)

^a^Modifier; age >55 years, pregnancy or comorbidity; *CI* confidence interval

Table 5 shows transport destinations for potential TSCI patients. A proportion of 29.9 % (95 % CI: 29.6–30.2) of all patients were transported to a MTS.

**Table 5:** Patient’s transport destination in Victoria, Australia (2007–2012)

Destination	*N* = 106060 (%; 95 % CI)
MTS	31727 (29.9; 29.6–30.2)
Specialised spinal centre	4646 (4.4; 4.3–4.5)
Other destinations	68250 (64.4; 64.1–64.6)
Unknown	1436 (1.4; 1.3–1.4)

*MTS* major trauma service, *CI* confidence interval

## Discussion

This retrospective cohort study is the first in Australia to describe the epidemiology of patients at risk of, or suspected to have sustained a TSCI presenting to paramedics. The overall median (IQR) age was 51 years (29–78) with an average incidence rate of 507 per million persons per year. Overall, patients with potential TSCI were more likely to be males, which is consistent with the published reports regarding confirmed TSCI (Pickett et al. 2006; Kattail et al. 2009; Norton 2007).

Falls were the most common cause of injury in those at risk of TSCI, which is also consistent with some previous studies of confirmed TSCI (Kattail et al. 2009; Celani et al. 2001; Pickett et al. 2003). This may be explained by the large proportion of patients aged older than 65 years included in our cohort (Table 3). The increased rates of low falls among elderly people may be related to various physiological changes associated with aging such as loss or decrease in eyesight, balance disturbance, slower reaction time, other neurological problems and reduced muscle strength (Muangpaisan et al. 2015). High falls tended to occur at a younger age and could be due to occupational or non-occupational incidents and include falls from ladders, roofs, trees, as well as scaffolding (Mitra et al. 2007). Importantly, these results point to the need for different falls prevention strategies in those at-risk of having low versus high falls due to the different circumstances, settings and age groups, respectively.

Traffic-related injuries were the second most common cause, contrary to several older studies reporting MVCs as the primary aetiology (Pickett et al. 2006; Sekhon and Fehlings 2001; O’Connor 2002; Burton et al. 2005). Motor Vehicle Collisions have been reported to generally involve males of all ages (Zimmerman et al. 2012; Majdzadeh et al. 2011; Rakotonirainy et al. 2012). Our results, however, show that MVCs were almost equally represented in both genders, but involving a greater proportion of males at a younger age (<34 years). This indicates that both males and females may be at equal risk of sustaining a TSCI as a result of a MVC, but with higher risk among younger males. It has been reported that young males have a greater propensity for risk-taking behaviours, thus resulting in a higher involvement in motor vehicle collisions (Rakotonirainy et al. 2012).

Injuries related to violence, sporting accidents, being struck by an object, incidents involving animals, industrial accidents, diving, crush and hanging comprised a small percentage similar to other reports (Sekhon and Fehlings 2001; Ackery et al. 2004).

Similar to previous studies, anatomic injuries were the most common PMT criterion (Boyle et al. 2008) and the modified MOI was the least common (Cox et al. 2011). These studies also reported that males comprised themajority of patients who met the PMT criteria (Cox et al. 2011; Boyle et al. 2008). Other studies have included the MOI in their major trauma criteria without modification, i.e., without modifying co-morbidities, age or pregnancy. These studies reported that MOI was the most common parameter of PMT criteria influencing their triage protocols (Fitzharris et al. 2012; Ma et al. 1999; van Laarhoven et al. 2014). For this study, the potential time critical category was combined with modifiers, that is, co-morbidities, age or pregnancy, since the state’s Pre-hospital Major Trauma criteria include modifiers with mechanism of injury in its definition. Such modification aims to minimise trauma over-triage that may result by relying on MOI only (Cox et al. 2011).

This study includes a very large cohort of people with potential TSCI and provides background information that forms a baseline for future research, and a reference point for making policy changes to future practice. Based on the Victorian state population of 5.7 million, confirmed TSCI cases are estimated to have comprised less than 0.5 % of this cohort (ie. 15 cases per million population) (28), however, corroboration of TSCI would require data linkage with other sources, such as admitted patient hospital data, trauma registry or spinal unit records. Future studies are required to identify rates of actual TSCI among potentials for the different injury causes, mechanisms, co-morbid disease and demographic subgroups, and to compare the differences between these two groups, aiming to identify the predictors of TSCI in the prehospital settings. Future research is also needed to identify paramedics’ and/ or guidelines’ sensitivity and specificity to identify actual SCI.

### Limitations

Our data is derived from the electronic patient care reports (ePCRs) recorded by paramedics who operate in a challenging pre-hospital environment. Therefore, some valid cases may have been misreported (errors of omission or accuracy) and/or underreported (misclassification). Nevertheless, recoding of variables, such as injury cause, gender and age, was undertaken following review of the narrative case descriptions provided by the treating paramedics. Yet, removing cases with missing integral data might have resulted in excluding valid cases as well, which may lead to under-reporting potential and/or confirmed TSCI, who were managed in the pre-hospital settings.

The PMT coding relied on complete recording of vital signs, anatomic location of injury, mechanism of injury and co-morbidities. Vital signs and anatomic location of injuries are consistently well reported, however, mechanism of injury and co-morbidity are not. It is possible that a number of cases may have met the PMT based on mechanism of injury and co-morbidity, but were not included due to missing data. As a result, the proportion of PMT might have been under-reported, potentially underestimating the burden of potential and/or confirmed TSCI cases.

Comparison of our results to other studies was difficult, as studies using EMS data are scarce. However, where available, studies using trauma registries or Emergency Department registries were used to compare patients’ demographic as well as injury characteristics.

## Conclusion

This is the first study to describe the epidemiology of potential TSCI in Australia and is based on a large, state-wide population registry. Falls and traffic related events were the most common causes of injury resulting in potential SCI. A proportion of 29.9 % t of patients were transported to a major trauma service with transport-related incidents being the most common cause of these potential TSCI cases. Also, 48.8 % of patients met the PMT criteria with the majority of these being due to anatomical injuries. Future research is required to identify the proportion of confirmed TSCI among the potentials.

## Abbreviations

3 m: Three meters
ALS: Advanced life support
AV: Ambulance Victoria
EMS: Emergency Medical Services
ePCRs: electronic patient care reports
IQR: Interquartile range
MICA: Paramedics and mobile intensive care ambulance
MOI: Mechanism of injury
MTS: Major trauma service
N: Number of cases
NFS: Not further specified
PMT: Pre-hospital major trauma
TSCI: Traumatic spinal cord injuries
VACIS: Victorian Ambulance Clinical Information System
VSTR: The Victorian State Trauma Registry
